# The Future of Biomechanical Spine Research: Conception and Design of a Dynamic 3D Printed Cervical Myelography Phantom

**DOI:** 10.7759/cureus.4591

**Published:** 2019-05-03

**Authors:** William Clifton, Eric Nottmeier, Aaron Damon, Conrad Dove, Mark Pichelmann

**Affiliations:** 1 Neurosurgery, Mayo Clinic, Jacksonville, USA; 2 Neurosurgery, Mayo Clinic, Rochester, USA

**Keywords:** 3d printing, spine, biomechanics, spine biomechanics, cervical, fluoroscopy, phantom design, phantom design, myelography

## Abstract

Background

Three-dimensional (3D) printing is a growing practice in the medical community for patient care and trainee education as well as production of equipment and devices. The development of functional models to replicate physiologic systems of human tissue has also been explored, although to a lesser degree. Specifically, the design of 3D printed phantoms that possess comparable biomechanical properties to human cervical vertebrae is an underdeveloped area of spine research. In order to investigate the functional uses of cervical 3D printed models for replicating the complex physiologic and biomechanical properties of the human subaxial cervical spine, our institution has created a prototype that accurately reflects these properties and provides a novel method of assessing spinal canal dimensions using simulated myelography. To our knowledge, this is the first 3D printed phantom created to study these parameters.

Materials and methods

A de-identified cervical spine computed tomography imaging file was segmented using threshold modulation in 3D Slicer software. The subaxial vertebrae (C3-C7) of the scan were individualized by separating the facet joint spaces and uncovertebral joints within the software in order to create individual stereolithography (STL) files. Each individual vertebra was printed on an Ultimaker S5 dual-extrusion printer using white “tough” polylactic acid filament. A human cadaveric subaxial cervical spine was harvested to provide a control for our experiment. Both models were assessed and compared in flexion and extension dynamic motion grossly and fluoroscopically. The maximum angles of deformation on X-ray imaging were recorded using DICOM (Digital Imaging and Communications in Medicine) viewing software. In order to compare the ability to assess canal dimensions of the models using fluoroscopic imaging, a myelography simulation was designed.

Results

The cervical phantom demonstrated excellent ability to resist deformation in flexion and extension positions, attributed to the high quality of initial segmentation. The gross and fluoroscopic dynamic movement of the phantom was analogous to the cadaver model. The myelography simulator adequately demonstrated the canal dimensions in static and dynamic positions for both models. Pertinent anatomic landmarks were able to be effectively visualized for assessment of canal measurements for sagittal and transverse dimensions.

Conclusions

By utilizing the latest technologies in DICOM segmentation and 3D printing, our institution has created the first cervical myelography phantom for biomechanical evaluation and trainee instruction. By combining new technologies with anatomical knowledge, quality 3D printing shows great promise in becoming a standard player in the future of spinal biomechanical research.

## Introduction

Three-dimensional (3D) printing has had an emerging presence in the medical field over the last decade [1,2]. The most common applications of this technology are patient education, preoperative planning, and trainee instruction both in procedural and anatomic learning. The functional applications of 3D printing have had some exploration with respect to creating models for the purposes of studying the physiologic properties of synthetic materials compared with human tissue [3,4]. The implications of discoveries from research in this field are of paramount importance for the development of models that resemble human tissue both in aesthetic qualities as well as functional and biomechanical abilities. Fused deposition modeling (FDM) 3D printing has numerous advantages for creating phantoms of human bone for radiologic and functional purposes. There is currently a disparity in the literature regarding the development of cervical models using FDM technology for these purposes [2,4-6]. 3D printing hardware by itself does not create accurately represented models. This is performed through “slicing”, or software development of virtual files that have desired properties according to their purpose. Quality is a factor of paramount importance in creating models with complex features, such as human vertebrae. This subjective component of 3D printed model production is a pivotal factor in determining the utility of a finished product. In order to investigate the functional uses of cervical 3D printed models for replicating the complex physiologic and biomechanical properties of the human subaxial cervical spine, our institution has created a prototype that accurately reflects these properties through open access high definition software analysis, and provides a novel method of assessing spinal canal dimensions using simulated myelography. To our knowledge, this is the first 3D printed cervical phantom created to study these parameters.

## Materials and methods

Phantom design and conception

**Figure 1:**
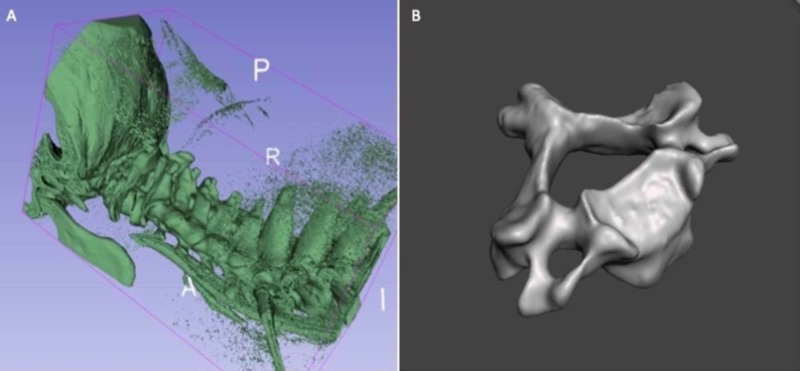
Software phantom design. (A) Image conversion to a stereolithography (STL) file using 3D Slicer. (B) Editing of the converted STL file to accurately represent the bony anatomical structure of the subaxial vertebrae.

A cervical spine computed tomography (CT) scan from a young male patient with no known cervical pathology was acquired and de-identified. This DICOM (Digital Imaging and Communications in Medicine) file was segmented using threshold modulation in 3D Slicer software. The subaxial vertebrae (C3-C7) of the scan were carefully individualized by separating the facet joint spaces and uncovertebral joints within the software in order to create individual stereolithography (STL) files for each vertebra (Figure 1). This key step was performed in order to deliver dynamic motion ability once the subaxial vertebral column was assembled post-production. Each STL file of the individual subaxial vertebrae was uploaded into a STL editing software (Meshmixer) for pre-production evaluation and then sliced using Cura software (Figure 1). Each individual vertebra was printed on an Ultimaker S5 dual-extrusion FDM printer using white “tough” PLA (polylactic acid) filament (10% infill) at a bed print temperature of 80 degrees Centigrade and nozzle temperature of 220 degrees Centigrade. After production the individual vertebrae were then assembled in anatomic order and quality checked for proper integration of the facet and uncovertebral joint spaces in anatomic orientation.

Phantom and control model dynamic assessment

After the assembled phantom was assessed for external quality, its ability to display flexion and extension within physiologic parameters was evaluated. Since the model did not contain a replacement for the subaxial discoligamentous complex, the dynamic motion assessed was based purely on the bony anatomical ultrastructure.

In order to provide a control specimen and validate the findings of our cervical phantom, we compared the dynamic properties and radiographic parameters of our model to a human cadaveric subaxial cervical spine. The C3-C7 vertebrae were harvested from a fresh cadaveric specimen and stripped of the ligaments and intervertebral discs using a combination of electrocautery and sharp dissection. The individual vertebrae were then soaked in enzymatic solvent to remove the excess tissue and inserted into a heated force cleaning cycle at 70 degrees Centigrade. Satisfied with complete soft tissue removal, the individual vertebrae were assembled in anatomic fashion. This specimen was used to compare flexion and extension dynamic motion to the cervical phantom. Measurements were taken using DICOM software (Horos) of the maximum degrees of flexion and extension for both models. These angles were determined by the intersection of the two lines drawn perpendicular to the endplates of C3 and C7 in maximum deformation before model failure. These angles were based on previously published parameters [7, 8]. Failure was defined as radiographic subluxation of vertebral bodies during manipulation.

**Figure 2:**
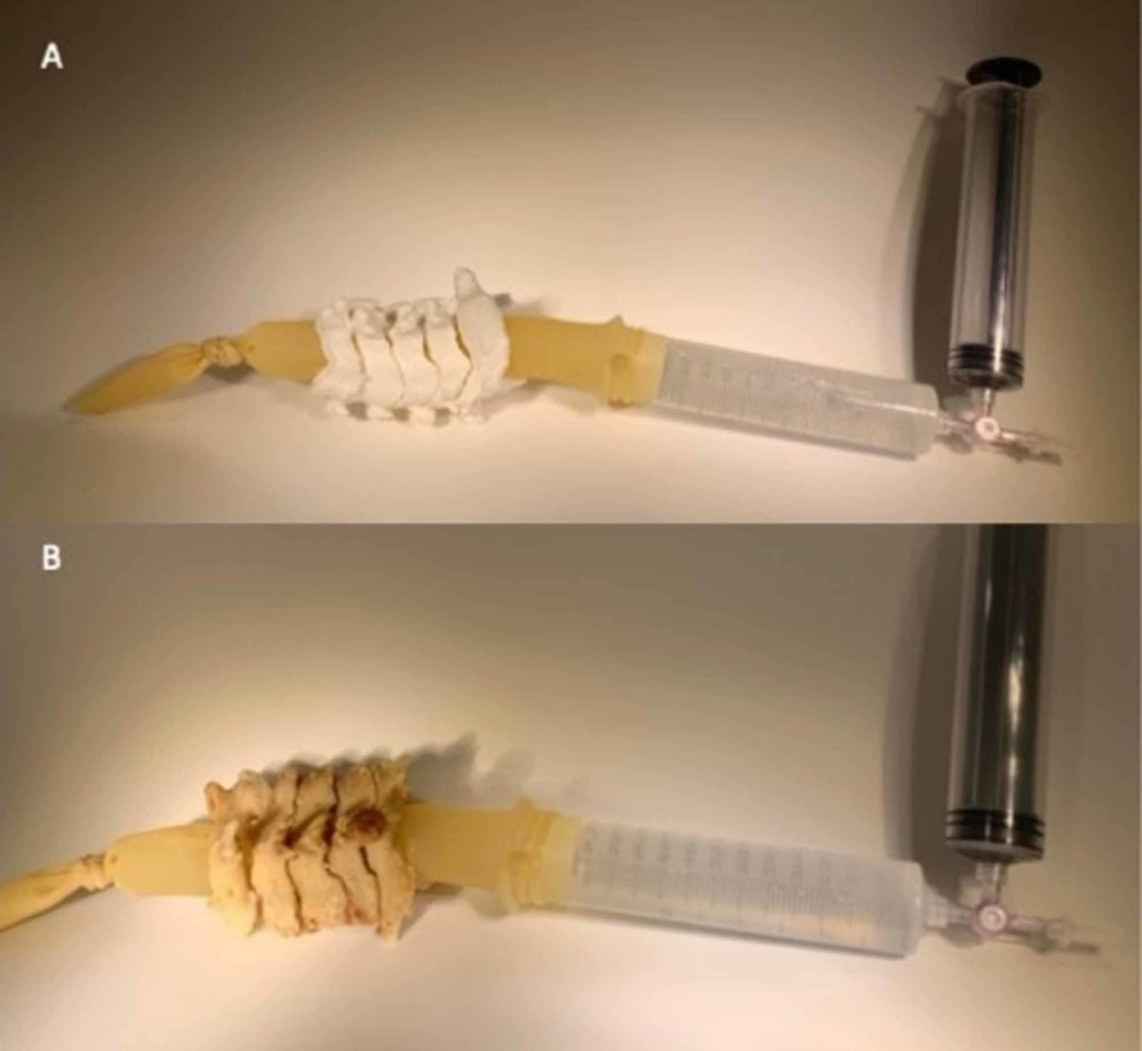
Assembly of myelography simulation. The myelography apparatus was inserted into the spinal canal of the phantom (A) and cadaveric specimen (B) in order to investigate the ability to quantify the spinal canal dimensions.

In order to assess canal dimensions of the model using fluoroscopic imaging, a myelography simulation was designed. Our first step was to recreate the contained subarachnoid space within the model spinal canal to allow for liquid contrast to fill the canal completely. This was accomplished by the use of an 18” x 1” latex Penrose drain. The drain was tied at one end and was then fixed to a 50 milliliter (ml) syringe connected to a second 50 ml syringe via a three-way stopcock. This allowed for modulated “filling” of the contrast during radiographic assessment. The system was then filled with Omnipaque and saline in a 1:9 ratio with a total system volume of 100 ml. This was successfully inserted through the spinal canal of the phantom and examined under fluoroscopy (Figure 2). The myelography simulator system was also placed successfully within the cadaveric spinal canal and examined under fluoroscopy.

## Results

Dynamic evaluation

**Figure 3:**
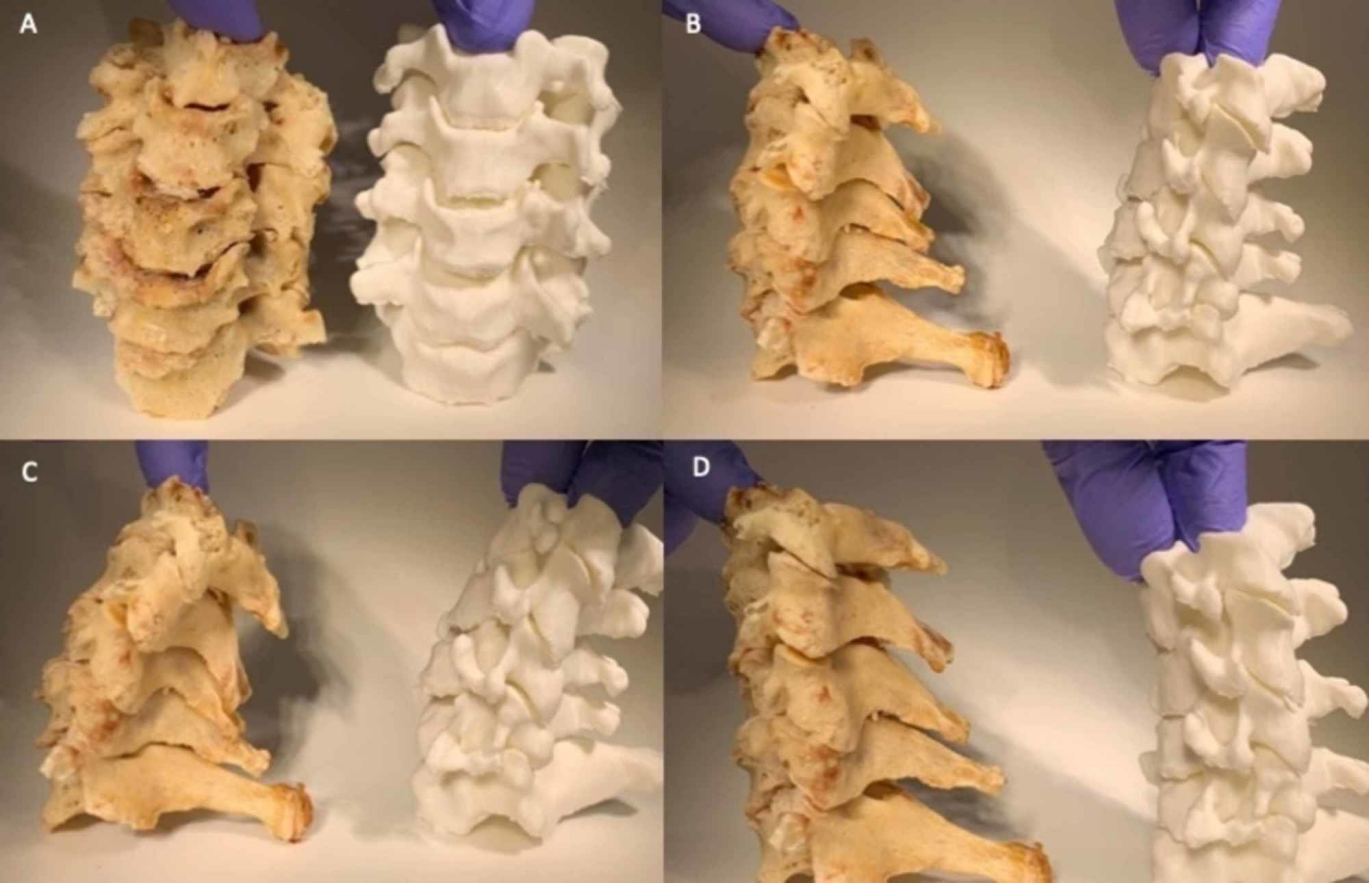
Dynamic comparison of cadaveric specimen to cervical phantom. The cadaveric specimen is located on the left in each frame, and the phantom on the right. (A&B) Neutral, (C) extension, and (D) flexion comparison of the models during external manipulation revealed comparable deformation parameters.

**Figure 4:**
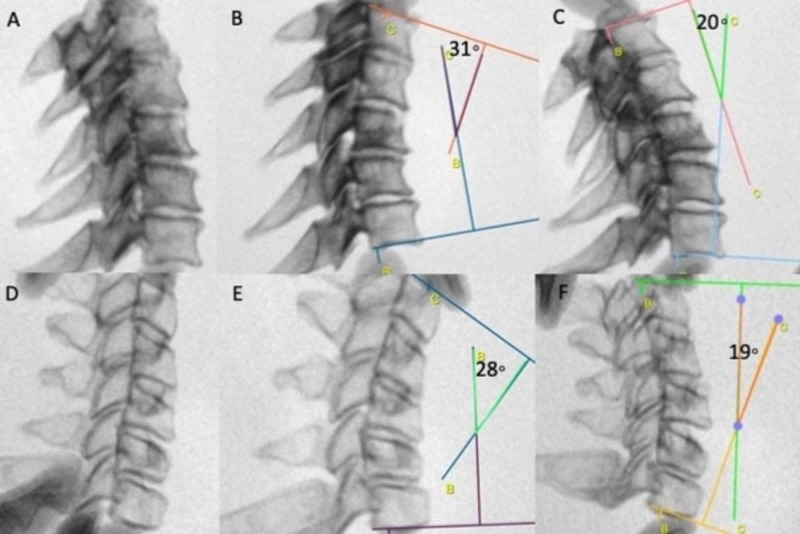
Radiographic comparison of the cadaveric control and phantom prototype to assess dynamic parameters. (A) Static, (B) flexion, and (C) extension evaluation under fluoroscopy of the cadaveric model demonstrated the ability to quantify the degrees of maximum flexion and extension. (D-F) Analogous static and dynamic evaluation of the 3D printed phantom demonstrated nearly identical angles of flexion and extension under direct manipulation compared with the cadaveric control.

The cervical phantom demonstrated excellent ability to resist deformation in flexion and extension positions with only bony anatomical components (Figure 3). This was attributed to the high quality of initial DICOM segmentation and detailed preservation of the facet anatomy and uncovertebral joint boundaries during production. The gross dynamic movement of the phantom was analogous to the cadaver model. The maximum degrees of flexion for the phantom and cadaveric models were 28 degrees and 31 degrees, respectfully. The maximum degrees of extension were 19 degrees and 20 degrees, respectfully (Figure 4). These measurements indicated restrictive values compared to normative data in previous population studies, however with almost equal values between the two models [7,9,10]. The lack of the discoligamentous complex in both models likely contributed to these values.

Myelography simulation evaluation

**Figure 5:**
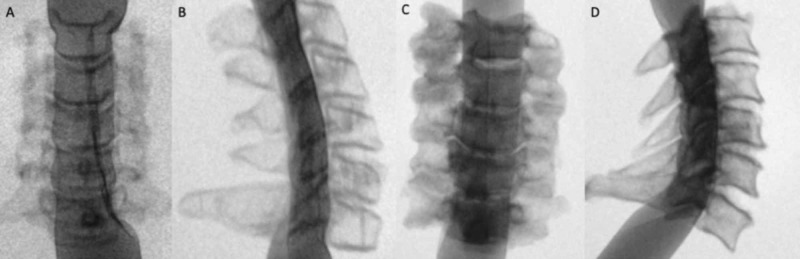
Fluoroscopic evaluation of myelography simulation. (A) Transverse and (B) sagittal images of cervical phantom after insertion of contrast agent into spinal canal through constructed apparatus. (C) Transverse and (D) sagittal images of cadaveric control. The spinal canal dimensions of both models were able to be assessed using the myelography simulator.

The myelography simulator was able to be inserted successfully in both the cadaver and phantom models. The Penrose drain filled the spinal canal in both models when inflated with the contrast/saline solution. The simulator adequately demonstrated the canal dimensions in static and dynamic positions for both models using our 9:1 ratio of saline:contrast. Pertinent anatomic landmarks were able to be effectively visualized for assessment of canal measurements for sagittal and transverse dimensions (Figure 5).

## Discussion

3D printed spine phantoms for biomechanical research are a powerful tool for assessing bony parameters of human vertebrae. FDM technology allows for detailed printing of patient-specific anatomical details of bony structure both for physiologic and pathologic conditions [2,11-14]. Cervical models have had limited investigations for biomechanical purposes in the current literature. A possible reason for this is the difficulty of reproducing the discoligamentous complex of the subaxial human vertebrae. Flexible 3D printing filaments have shown some promise in recreating the ligamentous complex of the human spine, however with variable results [1,13,14]. Reproduction of the bony-ligamentous complex is a difficult undertaking. However, quality reproductions of bony anatomy can be utilized for dynamic motion research utilizing fluoroscopic assessments. The subaxial cervical vertebrae have a complex bony structure to allow for flexion, extension, and lateral bending of the neck. Replicating detailed bony structure by 3D printing has its own challenges, namely accurate segmentation and production of the joint spaces both in the facets and the uncovertebral joints. This is able to be accomplished, however, through precise pre-production segmentation and STL editing [11,12]. Our current investigation shows that a quality modeling technique within appropriate software platforms is able to produce a prototype that demonstrates accurate motion across the individual vertebral segments in the subaxial cervical spine.

Our cervical phantom showed remarkable similarity to cadaveric tissue both in radiographic assessment and in dynamic motion. PLA was chosen for this initial prototype due to its durable properties and ease of printing. Other filaments with high hardness properties such as nylon, acryl butadiene styrene (ABS), and polypropylene (PP) may be used to create these models as well, however the radiographic appearance under fluoroscopy has yet to be fully explored for cervical spine phantoms [5]. The addition of myelography simulation to our model allows for evaluation of canal dimensions in both physiologic and extreme positions.

The development of this phantom has numerous applications for biomechanical testing and evaluation of 3D printed spine models. Degenerative or traumatic pathologies with spinal canal compromise such as ossification of the posterior longitudinal ligament, facet hypertrophy, vertebral body fractures or dislocations, and many other conditions can be assessed via this model. Patient-specific pathologies may also be investigated before surgical intervention using this system, such as canal changes during surgical positioning and dimensional alterations after decompression. This system is not necessarily limited to cervical spine use and may be employed in the lumbar, thoracic, and sacral spines as well. Creation of this system for 3D model evaluation opens many doors for the study of patient-specific models and related outcomes [13,15-17]. A limitation of our model is that it currently does not provide a substitute for cervical ligamentous tissue. As 3D printed models improve in the ability to replicate the ligament/bone interface, the need for cadaveric tissue for trainee education or biomechanical purposes will decrease [17-20].

## Conclusions

By utilizing the latest technologies in DICOM segmentation and 3D printing, our institution has created the first cervical myelography phantom for biomechanical evaluation and trainee instruction. The elementary dynamic parameters of this model compared strikingly to cadaveric tissue during manipulation and fluoroscopic evaluation. Application studies of this concept are currently ongoing to determine the efficacy in predicting patient-specific outcomes after surgical intervention, as well as trainee education and instruction. By combining new technologies with anatomical knowledge, FDM 3D printing shows great promise in becoming a standard player in the future of spinal biomechanical research.

## References

[1] Filippou V, Tsoumpas C (2018). Recent advances on the development of phantoms using 3D printing for imaging with CT, MRI, PET, SPECT, and ultrasound. Med Phys.

[2] Wu AM, Lin JL, Kwan KYH, Wang XY, Zhao J (2018). 3D-printing techniques in spine surgery: the future prospects and current challenges. Expert Rev Med Devices.

[3] Murgitroyd E, Madurska M, Gonzalez J, Watson A (2015). 3D digital anatomy modeling - practical or pretty?. Surgeon.

[4] Wilcox B, Mobbs RJ, Wu AM, Phan K (2017). Systematic review of 3D printing in spinal surgery: the current state of play. J Spine Surg.

[5] Bohl MA, Mooney MA, Repp GJ, Nakaji P, Chang S, Turner J, Kakarla U (2018). The Barrow biomimetic spine: fluoroscopic analysis of a synthetic spine model made of variable 3D-printed materials and print parameters. Spine.

[6] Bohl MA, Morgan CD, Mooney MA (2019). Biomechanical testing of a 3D-printed L5 vertebral body model. Cureus.

[7] Kim SW, Kim TH, Bok DH (2018). Analysis of cervical spine alignment in currently asymptomatic individuals: prevalence of kyphotic posture and its relationship with other spinopelvic parameters. Spine J.

[8] Reitman CA, Mauro KM, Nguyen L, Ziegler JM, Hipp JA (2005). Intervertebral motion between flexion and extension in asymptomatic individuals. Spine.

[9] Yu YL, du Boulay GH, Stevens JM, Kendall BE (1985). Morphology and measurements of the cervical spinal cord in computer-assisted myelography. Neuroradiology.

[10] Bogduk N, Mercer S (2000). Biomechanics of the cervical spine. I: normal kinematics. Clin Biomech.

[11] Szymor P, Kozakiewicz M, Olszewski R (2016). Accuracy of open-source software segmentation and paper-based printed three-dimensional models. J Craniomaxillofac Surg.

[12] Wu A-M, Shao Z-X, Wang J-S (2015). The accuracy of a method for printing three-dimensional spinal models. PloS ONE.

[13] Zhang F, Zhang H, Zhao H (2019). Design and fabrication of a personalized anthropomorphic phantom using 3D printing and tissue equivalent materials. Quant Imaging Med Surg.

[14] Leng S, McGee K, Morris J (2017). Anatomic modeling using 3D printing: quality assurance and optimization. 3D Print Med.

[15] Wu AM, Wang K, Wang JS, Chen CH, Yang XD, Ni WF, Hu YZ (2018). The addition of 3D printed models to enhance the teaching and learning of bone spatial anatomy and fractures for undergraduate students: a randomized controlled study. Ann Transl Med.

[16] Park HJ, Wang C, Choi KH, Kim HN (2018). Use of a life-size three-dimensional-printed spine model for pedicle screw instrumentation training. J Orthop Surg Res.

[17] Lim KH, Loo ZY, Goldie SJ, Adams JW, McMenamin PG (2016). Use of 3D printed models in medical education: a randomized control trial comparing 3D prints versus cadaveric materials for learning external cardiac anatomy. Anat Sci Educ.

[18] Huang H, Xiang C, Zeng C, Ouyang H, Wong KK, Huang W (2015). Patient-specific geometrical modeling of orthopedic structures with high efficiency and accuracy for finite element modeling and 3D printing. Australas Phys Eng Sci Med.

[19] Chang D, Tummala S, Sotero D (2019). Three-dimensional printing for procedure rehearsal/simulation/planning in interventional radiology. Tech Vasc Interv Radiol.

[20] Older J (2004). Anatomy: a must for teaching the next generation. Surgeon.

